# “All things equal”: ethical principles governing why autonomous vehicle experts change or retain their opinions in trolley problems—a qualitative study

**DOI:** 10.3389/frobt.2025.1544272

**Published:** 2025-05-27

**Authors:** Stephen R. Milford, B. Zara Malgir, Bernice S. Elger, David M. Shaw

**Affiliations:** ^1^ Institute for Biomedical Ethics, University of Basel, Basel, Switzerland; ^2^ Department of Theology, North-West University, Potchefstroom, South Africa; ^3^ Center for Legal Medicine, University of Geneva, Geneva, Switzerland; ^4^ Care and Public Health Research Institute, Maastricht University, Maastricht, Netherlands

**Keywords:** autonomous vehicle, expert opinion, trolley problem, crash scenario, ethics

## Abstract

**Introduction:**

Autonomous vehicles (AVs) are already being featured on some public roads. However, there is evidence suggesting that the general public remains particularly concerned and skeptical regarding the ethics of collision scenarios.

**Methods:**

This study presents the findings of the first qualitative research into the ethical opinions of experts responsible for the design, deployment, and regulation of AVs. A total of 46 experts were interviewed in this study and presented with two trolley-problem-like vignettes. The experts were asked for an initial opinion on the basis of which the parameters of the vignettes were changed to gauge the principles that would result in either changing or retaining an ethical opinion. Much research has been conducted on public opinion, but there are no available research findings on the ethical opinions of AV experts.

**Results:**

Following reflective thematic analysis, four important findings were deduced: 1) although the expert opinions are broadly utilitarian, they are nuanced in significant ways to focus on the impacts of collision scenarios on the community as a whole. 2) Obeying the rules of the road remains a significantly strong ethical opinion. 3) Responsibility and risk play important roles in how AVs should handle collision situations. 4) Egoistic opinions were present to a limited extent.

**Discussion:**

The findings show that the ethics of AVs still pose a serious challenge; furthermore, while utilitarianism appears to be a driving ethical principle on the surface, along with the need for both AVs and vulnerable road users to obey the rules, questions concerning community impacts and risk vs. responsibility remain strong influences among AV experts.

## 1 Introduction

Autonomous vehicles (AVs) promise to radically improve human lives. Aside from the 1.2 million estimated reduction in road deaths (Fagnant and Kara, 2015; Gao et al., 2016; Airbib and Seba, 2017; WHO, 2018), AVs promise to improve traffic control (Stead et al., 2017; Szele and Kisgyörgy, 2018), access for the disabled (Resnik and Andrews, 2023), economic benefits (Othman, 2022), and city environments (Kopelias et al., 2020). To realize these benefits, there must be widespread pubic adoption of AVs (Rowthorn, 2019; Nastjuk et al., 2020; Othman, 2022; Zhou et al., 2023); however, there is evidence suggesting that the general public has serious concerns regarding AV adoption (Faverio, 2022; Gross, 2022; Rainie et al., 2022; Rowthorn, 2019; Smith and Anderson, 2017). One of the important concerns for the public is the ethics involved in deploying AVs on public roads, particularly those entailing crash scenarios (Gill, 2021; Liljamo et al., 2018).

These scenarios are often presented in the form of trolley-like gambits that were first popularized by Foot (1967). Some researchers argue that modern technology can prevent all such occurrences or that such cases will be too rare to merit attention (Cunneen et al., 2020; Davnall, 2020; Hansson et al., 2021; Holstein and Dodig-Crnkovic, 2018; Lundgren, 2021; Schäffner, 2021). However, Evans et al. (2020) contend that even if rare, the “fact remains that lethal, serious and near-accidents will continue to occur.” Considering that millions of cars are presently sharing public roads globally and being driven billions of miles annually, life and death scenarios are bound to occur; this is particularly true in mixed traffic where AVs interact with numerous manned and unmanned road users (Awad et al., 2019; Bonnefon et al., 2016; Fleetwood, 2017; Formosa, 2022; JafariNaimi, 2018; Kopecky et al., 2023; Martinho et al., 2021; Millan-Blanquel et al., 2020; Nyholm and Smids, 2016; Robinson et al., 2022; Rowthorn, 2019; Wang et al., 2023). Unlike human drivers, AVs must be programmed to respond in such dilemma scenarios, and their decision making algorithms (DMAs) cannot be left unguided (Emerging Technology, 2015; JafariNaimi, 2018; Shaw and Schneble, 2021). However, the question as to how to program these DMAs remains open. Sizable amounts of normative and empirical works have already been undertaken, including exploration of public opinions on the relative importances of different features of characters within trolley problem scenarios (Awad et al., 2018; Gantsho, 2021; Hilgarter and Granig, 2020; Königs, 2023; Lawlor, 2022; Lazányi, 2023; Schneble and Shaw, 2021; Bleske-Rechek et al., 2010). Nevertheless, no empirical research efforts have been undertaken to consider the opinions of people and experts who actually design, deploy, and regulate such AVs. This is a significant oversight considering that it is these experts who are ultimately responsible for how the AVs will behave on public roads.

The findings presented herein represent the first empirical research into the ethical opinions of experts in the field of AV design, deployment, and regulation. Using well-established qualitative research methodologies, we interviewed 46 AV experts on the topic of the ethics of AVs in crash scenarios. By analyzing these data, we can gain an in-depth understanding of the ethical principles held by experts who ultimately influence the manner in which AVs are programmed, deployed, and regulated.

## 2 Methodology

### 2.1 Sample and data collection

As part of the Proactive Ethical Approach to Responsible Automation (PEpp) project within the NCCR (automation), a research center funded by the Swiss National Science Foundation, experts in the field of AV design, deployment, and regulation were interviewed. In total, 46 experts participated in this work. As this project was funded from the perspective of the Swiss context, a Swiss cohort (comprising 24 participants) and an international cohort (comprising 22 participants) was recruited. The participants were primarily from Western countries, such as Switzerland, Italy, Germany, the United Kingdom, and the United States of America, with some being of Asian (Chinese and Indian) background but working in Western contexts. Additional details are presented in the Limitations section. Of these participants, 33 experts had direct AV development experience, e.g., as the CEO of a private AV development company, working as AV testers, developing AV algorithms at prestigious universities, or being involved in AV trials on public roads. Of the remaining participants, 10 experts had general artificial intelligence or automation/control programming experience with AVs or traffic control, such as in traffic management, or shared mobility. The final three experts held positions in AV regulatory bodies in Switzerland, the European Union, and at the state level in the United States.

Following ethics approval, a semistructured interview guide was developed and piloted with three Swiss AV experts. The interview guide was then modified slightly in light of the pilot interviews. Given the research aims to explore the ethical opinions of specific expert populations, purposeful as well as snowball recruiting was conducted for the remaining participants (Blackstone, 2012; Marshall, 1996). The interviews were continued until saturation, which is defined as the point at which no new significant findings emerge from the interviews and the amassed qualitative data are sufficient to enable robust conclusion to be formulated, with both the Swiss and International cohorts; thereafter, two additional interviews were conducted in each cohort to confirm saturation (Saunders et al., 2018).

**FIGURE 1:**
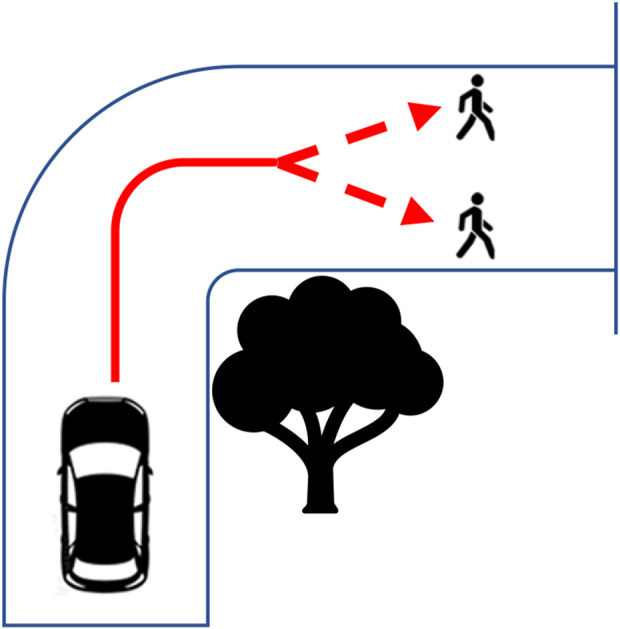
Vignette 1.

**FIGURE 2:**
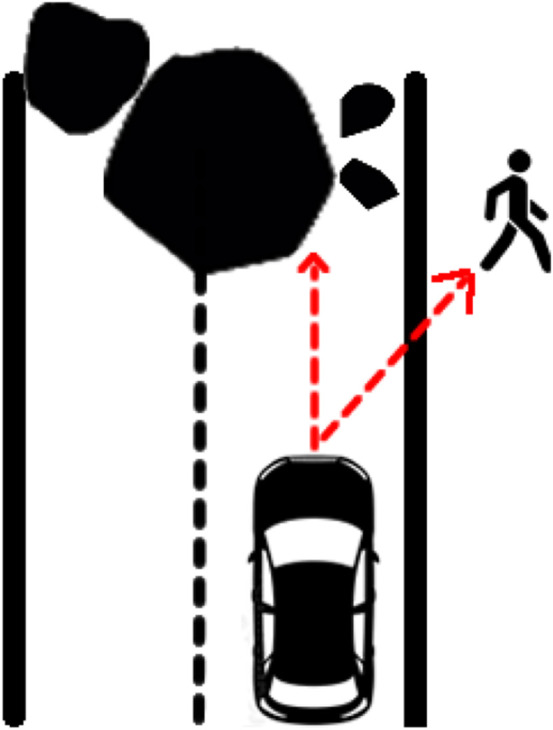
Vignette 2.

An experimental ethics approach to qualitative research was chosen for this study to encourage greater participant engagement. This method involves actively and softly challenging the participants so as to encourage active reflection and development of participant responses (Campbell and Kumar 2012; Kahane, 2013; Knobe et al., 2010). The participants were presented with two fictional vignettes in which an AV faced a no-win binary choice. Irrespective of the decision made by the AV, one or more persons would die. Vignette 1 (Figure 1) was designed with a neutral framing consideration by presenting an AV turning a blind bend only to find two neutral pedestrians (with no specific characteristics) standing on the road, where the AV must choose to hit one of the two. The participants were asked what they would like to know about the pedestrians in the vignette before programming the DMA response. This neutral framing enabled the participants to introduce features of the characters that they personally found to be relevant, rather than being led by a list of features presented by the interviewer. In Vignette 2 (Figure 2), the AV was presented as traveling on a road at speed when a boulder falls in its path unexpectedly; here, the AV has no choice but to crash into the boulder and kill the occupant or swerve onto the pavement and kill a pedestrian instead. This second vignette examines self-concern vs. concern for others as well as the implied responsibility of an AV on public roads.

Inspired by the experimental philosophical bioethics methodology, the parameters of the vignettes were modified in response to the answers given by the participants (Earp et al., 2020). Initially, the participants were asked about what they thought the AV should do in either case without being provided any further clarifications regarding the nature or features of the characters involved in the fictional examples. It was assumed that the participants would provide unique responses to these initial presentations. As such, the follow-up challenges were not systematic or predefined but emerged naturally in response to the initial answers given by the participants. For example, a participant may say that the AV in Vignette 1 should make no alteration to its course on the basis that one of the fictional characters was on the wrong side of the road, or a participant may state that the AV in Vignette 2 should leave the road and strike the pedestrian on the basis that cars have a duty to protect their owners. In response to the initial answers given by the participants, the interviewers gently probed the participants in an attempt to change their opinion by amending the features of the fictional characters (occupants and pedestrians), such as adding more characters (to one side of the road, inside the car, or on the pavement), changing the age of a character, suggesting a unique state to one of the characters (e.g., being pregnant), or highlighting that one character is on the wrong side of the road. The participants were then encouraged to reassess their initial responses in light of these amendments and provide reasons for either retaining or modifying their answers.

This flexible approach intended to avoid consistency bias as well as order or framing bias by flexibly changing the features of the characters such that each participant was presented with a unique follow-up prompt depending on their original responses with the aim of exploring the underlying reasons as to why they would either retain or change their opinions when challenged.

### 2.2 Data analysis

The interviews were audio recorded and transcribed verbatim by the research team. All personally identifying data (name, place of work, role, etc.) were removed, and the anonymized transcripts were imported into MAXQDA—a standard program for qualitative research analysis (VERBI Software, 2024). The data were subjected to reflexive applied thematic analysis, a well-established qualitative research methodology, with the aim of analyzing and highlighting thematic elements to describe and interpret a dataset (Braun and Clarke, 2006; Guest et al., 2012; Mayring, 2014). This method involves five steps. In Step 1, the researchers familiarized themselves with the data by actively reading transcripts while noting some initial ideas for coding and themes. Step 2 involved open coding, whereby descriptive codes were inductively generated from the data. Three transcripts were initially coded, following which the principal investigator and research team met to evaluate the coding and initial code tree. Once the team agreed on these parameters, the remaining transcripts were coded through regular meetings for quality assessments. The code tree continued to evolve until reaching saturation. In Step 3, the overarching themes were identified by sorting the descriptive codes into groupable units. These themes were presented for review during the research team meeting in Step 4 to critically reflect upon as well as create new themes and codes. In Step 5, the themes were interpreted and data extracts were organized into coherent accounts supporting the core narratives present in the data.

## 3 Results

The study participants displayed a range of opinions when their initial responses were challenged by changing the parameters of the vignettes. These responses can be grouped into five broad categories as follows: complete change of opinion for both vignettes (8 participants); no change of opinion for either vignette (13 participants); change in opinion for Vignette 1 but not 2 (6 participants); change in opinion for Vignette 2 but not 1 (2 participants); possible change but uncommitted to the new position (11 participants). In the last category, the participants expressed a change or an uncertainty about their original opinion but did not display commitment to their new or changed positions. As an example, the following transcript shows the response by participant 12:

Like saving a school bus of children sounds like, way better, especially from … the company's objectives, a company PR perspective. That you're definitely probably going to want to save the school bus over that one person … actually, that's still really hard. I don't even know … because you're still killing one person. That doesn't sound good either. This is not good either way for the company.

These five broad groupings were unsurprising by themselves as they represent all possible responses. However, several significant themes emerged from the data as to why the participants either changed or retained some or all of their initial opinions. Some of these themes also crossed over between responses. For example, a utilitarian position was used to either justify a change of opinion (e.g., p#14 and 21) or reaffirm an original position in the face of changing parameters (e.g., p#11, 15, 31, and 36). Themes 1, 2, and 3 presented below were held by a significant number of participants; although Themes 4, 5 were held by a small number of experts, they were significant enough to merit mentioning.

No rich data were obtained from six of the participants, whose responses were evasive and deflective or avoidant for answering the question, even when softly pressed. Some examples of these responses are shown below:

I have no opinions here because I don't think that there is a correct answer … if you cannot identify anything, throw a coin. What I’m saying is if the technology is not at the level to throw coins very rarely, stop the technology. (p#28)

It is possible that I brake in time [so] that I don't hit the boulder … as a matter of fact … there is a horizon, there is a steering, breaking … all of those are continuous. (p#2)

… The distance is relevant. I mean, the distance is an option in consideration, then indeed you would steer away from the closer one because it would give you more time to stop … (p#22)

The car does not only have these two options. I want the car to make a decision that is based on risk distribution and takes into account several principles. (p#6)

### 3.1 Theme 1: numbers are important

A significant proportion of the participants (17) referred to a utilitarian view as the basis for either changing or not changing their opinions. Some participants, such as participant 18, used the term “utilitarianism” expressly, while others simply referred to saving more lives as the key motivator. For some participants such as participants 8, 12, and 18, this was a clear choice rooted in simply saving more lives. As per these participants, as long as all lives were “equal,” an AV should attempt to save as many lives as possible. In the case of participant 44, the exact balance between the numbers was not easy to express, as shown below:

Would you rather save 1,000 people or one person? You can probably go for the 1,000, wouldn't you? … there's not a very strong rational reason for saying if it's just two to one that you wouldn't go for the two rather than the one. (p#44)

For other experts like participants 12 and 14, a preference for a utilitarian point of view was rooted in pragmatism; that is to say, they felt it was difficult to program an AV to predict the movements of a group of people. Therefore, striking a single person was easier to program and resulted in a more predictable outcome:

… I'd want to look at the … movement of the people. If there's any chance of reducing the severity of the collision, then I would go in that direction. Otherwise, you know, I think then yes, if all things equal, I would say [hit] the individual over the multiple people. (p#12)

… I think there’s also an argument, and a very reasonable argument, that it's much harder to predict the actions and movements of a group of people than it is of a single person. Therefore, you are better off steering towards the single person because you can make better predictions about what they’re likely to do and so there is a reasonable case [to be made] that it is safer to give more space to the group than it is to the individual. (p#14)

**Table:** 

Data extract: the importance of numbers	
Change of opinion	• … saving a school bus of children sounds like way better … you’re definitely probably going to want to save the school bus over that one person. (p#9) • I think then yes, if all things equal, I would say [kill] the individual over the multiple people. (p#12) • If you’re considering an action which is likely to kill a number of civilians or has the possibility of doing it and the number is greater in one course of action than in the other, then that would definitely be a factor that you take into account. (p#19) • I guess you’d go for less rather than hitting more. You want to make the least impact possible. (p#21) • If I have to hit someone, I will hit the [side] of fewer people. (p#41) • I think if you statistically can determine if I do this then I’m gonna kill two people versus one, then I think it would make logical sense … to try to minimize the loss of life. (p#43) • … essentially, we wanna minimize damage [number of lives]. (p#44) • Now, if indeed it is an automated vehicle [that] is fast enough to detect those 10 people, then I think it probably should minimize the number of [deaths]. (p#47)
No change of opinion	• It’s the same as should you kill one person to save a hundred people in a hospital. Now, our ethics is not allowing that. You have a healthy person, should you remove all [of] his or her organs to save ten people? Are we implementing this in reality? The human ethic in medical care has decided that this is not ok. (p#36)
Uncommitted to the change of opinion	• … if there is a crowd of people, you’re not going to collide with them all the same as you would with the one person, so I feel like there’s a lot of other things that make it very hard for me to reason about this. And even if there is like a crowd of people, it’s probably worst to barrel into that. I think most people will agree that’s not good, but there’s so many little things that maybe it would be better to hit just the one person … (p#5) • Somewhere in my head, there is a limit at one point. If a bus needs to kill a person to save everybody, that would at some point make sense, I guess. But I think for me, this bar is pretty high. They had to land a plane to save all the passengers … by doing that, they crashed a boat with a fisherman or something. That’s ok. I guess it was for the best. (p#8) • I don't know. I mean, I would, this is, I mean like, again, it’s just one occupant in the car, one occupant and the person. What do you know about the person in the car? What do you know about the person who’s walking around you? … I can’t decide. I mean, if they’re like five people in the car, perhaps, you know, like, you should run over the pedestrian. But if it’s just some random dude, • I do gravitate towards that [utilitarianism] when all other things are equal. But I’m nowhere near. I’ve not thought about this enough to know what those numbers and trade-offs would be, but all other things being equal, yes, I would prefer to do less harm, I believe. (p#18) • Everything being said, then a smaller group would be less painful than a bigger group to me. I mean that’s somehow obvious. (p#35) • … probably then it would be the [one] person. I would just see the number of lives that are at stake. (p#37) • I have no answer. I don't know. Maybe, you can draw the worst scenario, which is like there’re 20 school kids waiting for a bus. I don't know. I mean … if that’s a thought experiment, it’s also not clear that you really can segment it that well. Sometimes you don't even know [how many people are involved]. (p#48)

### 3.2 Theme 2: rules are important

Among the experts, 10 participants considered the notion of obeying the rules to be of paramount importance. This was particularly evident for those whose opinions changed when the parameters of Vignette 1 changed. For example, it was noted that one of the pedestrians may be on the “wrong” side of the road in Vignette 1. This was particularly evident in the case of four participants who declined to change their opinions when the parameters of Vignette 2 were changed (e.g., adding more occupants to the AV). Although participant 41 argued that the very concept of “wrong side” (jaywalking) was “one of the most unethical things” because it “strips people of their own public space,” the other experts considered this to be a significant factor in deciding the behavior of an AV. According to participants 15 and 36, there are well-established rules of the road and that AVs cannot simply “invent” (p#15) new rules because they are newer technologies; as per participant 36, “[there is] nothing we can do. We will not decide to kill people that had the right to be there” (p#36) in the context of a pedestrian who was on the correct side of the road.

Likewise, participant 31 (speaking about Vignette 2) argued that it is against the rules of the road for a car to mount the pavement: “roads are for cars, and the pedestrian way is for pedestrians … to me, it seems like quite clear the car should actually stick to the road.” According to participant 31, even if this meant a passenger (or more) would be killed, the “pedestrian has nothing to do with it.” These respondents were very much committed to their opinion and went on to argue that “machines would decide better than humans” in this respect because the “machine actually obeys the rules.” Participant 11 contended that this was an issue of safety; if the car were to swerve (into another lane or onto the pavement) “then goodness knows, you’re pulling into a worse [situation].”

According to participant 16, both the programmer and pedestrians should obey the rules; they argued that they would not program a car to cross a double yellow line to strike someone “in a lane that [they] should not be in” when referring to the car being on the wrong side of the road. Consequently, they would program the car to “strike the person that’s doing something illegal [being on the wrong side].” While participant 11 agreed with this notion, they added that if cars were programmed to break the rules at any point, it could lead to numerous scenarios in which the car might break the rules:

If you tell a machine that what you should do is stay on the road, stay between the white lines in all circumstances, then you’re not giving any flexibility. You’re not saying unless there is this, this, this, and this, because you'll soon run out of lists…

### 3.3 Theme 3: responsibility is important

With regard to the theme of obeying the rules, 10 participants referred to the notion of who bore the most responsibility or risk. For example, participant 5 was quick to change their opinion for Vignette 1 when it was noted that one of the characters may be on the wrong side of the road; participant 5 argued that “there is some responsibility on the people to be … not jaywalking.” They argued that a person would be at fault if they were in a place that they were “not expected [to] be,” while participant 6 concurred by arguing that someone who was on the wrong side of the road “bears responsibility to avoid that crash” and therefore “responsibility should be one principle next to others [principles].”

However, the notion of responsibility was particularly strong among some of the participants (8 experts) for not changing their initial opinions for Vignette 2. Regardless of how the parameters of the vignette changed (more lives, ages of the victims, etc.), these participants held fast to the notion that the occupant of the AV “has decided to take the risk of traveling … and the person walking to the side didn’t make that decision” (p#14); therefore, it was the “responsibility of the vehicle to do everything it can to avoid … harming others.” This was true even for children who were occupants of an AV “because the parents want the kids to take the [AV], they [parents] must accept that they [children]… are exposed to dangers” (p#45). According to participant 48, this responsibility was the reason they were not fully committed to a utilitarian perspective (see table above). They argued that the “people using the service [AV]… are signing up for more risk than people just … going about their daily life.”

### 3.4 Theme 4: being good is important

Although this theme did not include a large number of participants, five participants noted the relative value between people based on their character as the reason for changing their opinions. This was most clearly expressed by participant 42 as follows:

… if there's only one factor that should bias my decision, it will be essentially how valuable is any of those to society … would somebody miss them or not? How desperately would they be missed … an old person can be a great contributor in whatever they're doing in life and a young person could be the opposite. So, it's definitely a factor … if there's only one thing I should know, then it would be that one. How valuable are they to society?

Similarly, participant 17 asked “what has the person achieved? Ultimately, you know, are they a good person? Are they a bad person?” Although participant 18’s first reaction was to ask “whether they’re [pedestrian] good people,” they were open to a utilitarianist approach of “all other things being equal” (see table above) but added a caveat by stating that they would choose “one delightful individual” over “five horrible, nasty serial killer[s].” They went on to state “if it was Nelson Mandela versus Charles Manson, you would probably choose Charles Manson.” Participant 21 argued that “if you have to choose between … a good person and a pedophile, then … hit the pedophile.”

### 3.5 Theme 5: self-preservation is important

In addition to the characters of the people involved, three participants insisted that protecting the occupant of an AV was fundamentally important and that they would not be dissuaded by any changes in the parameters of Vignette 2 (adding more pedestrians, changing ages, etc.).

… you [as a driver] will totally ignore that there are other people left and right, I will try to avoid the rock, self-preservation and that's all. I wouldn't be going further than that. And [just] as we accept something for the human driver, we should accept the same thing that will happen with the autonomous driver. There are drivers who will say “I prefer suicide” … and others who say “it's not my problem, I have to survive.” Whom you will accuse? In either case, the driver will not be accused. (p#25)

I think the machine has to act in a way to save the people sitting in the vehicle … no matter what, that comes first. So, I think from a developer point of view, and that will be the direction they [developers/manufacturers] will go with [this], the machine would hit the person … I think that that is something I've heard from different companies. The highest point for them is [that] the people within the vehicle have to survive … I think you do everything, whatever you think [is] possible for you [occupant] to survive. (p#46)

## 4 Discussion

### 4.1 Rejection of simplistic utilitarianism

The novelty in the findings presented above is the considerable nuance presented by a number of participants who appeared to hold utilitarian positions. However, the fact that such a large number of participants mentioned utilitarian principles as important reasons to either change or retain their opinions is not novel in itself. Utilitarianism is widely discussed in the context of AV trolley problems and often juxtaposed with deontological positions (Geisslinger et al., 2021; Jenkins et al., 2022; Liu and Liu, 2021; Sommaggio and Marchiori, 2020). Empirical research has shown that large sections of the general public are in favor of saving more lives than fewer (Frison et al., 2016; Awad et al., 2018; Bonnefon et al., 2016). Indeed, it has been argued that this may be incorporated in AV DMAs successfully (Geisslinger et al., 2021). In this regard, many of the participants of this study appeared to affirm the findings of the empirical research on public opinions as well as the ethical dilemmas arising from AVs and trolley problems by promoting a utilitarian perspective.

However, some participants who represented Theme 1 were inclined to reject a simplistic understanding of utilitarianism as saving more lives than fewer. For example, participant 8 was reluctant to save only a few lives over a single life, arguing that the bar should be set quite high; they connected this reluctance to the responsibility assumed by those driving AVs while pedestrians assume no such risks. As such, one needed to balance the responsibility assumed with the number of people at risk. Participant 12 noted that utilitarianism was only applicable “if all things [were] equal.” In this regard, they noted the complexity of trolley problems and challenged the simplistic understanding of utilitarianism. Real life very rarely presents as “all things being equal.” Participant 12 as well as other experts like participants 5, 14, and 21 appeared to rely on pragmatic rather than philosophical reasons to support a utilitarian view. For example, it would be harder to predict the movements of a group of people rather than a single person; therefore, groups should be avoided as a general rule for safety reasons. On the other hand, participant 5 noted that it might be beneficial to aim for the group as one would not collide with every member in the group, thereby distributing risk. However, it should be noted that participant 5 was not in favor of generally programming AVs to barrel through crowds.

It is interesting to note that some of those who referenced a utilitarian perspective may have done so for the same reasons as participants who focused on the importance of good character. Some participants in the utilitarian cohort spoke of the “least impact possible” (p#21), what “would be less painful” (p#35), or “limiting damage” (p#44) in collision scenarios. Such statements had the connotation of focusing on the impact, damage, or pain experienced by the community; in other words, the focus is on minimizing harm rather than explicitly maximizing benefit, which is very much like the cohort of participants in Theme 4, who noted the value of good character. Here, the implications for the community regarding killing a good person versus a bad person were emphasized rather than the number of people killed. The focus of both cohorts (utilitarian and good character) was on the impact of death on a community. This represents a more nuanced understanding of utilitarianism than that represented in quantitative studies, such as The Moral Machine Experiment (Awad et al., 2018). Rather than simply counting the lives lost, the utilitarian views in our study were sometimes strongly connected with the welfare of the community. Thus, although utilitarian principles were by far the most significant reasons in this research for changing or not changing the initial opinions, the reasons behind the utilitarian appeal were wide-ranging and nuanced.

In their nuanced utilitarian approach, the participants whose opinions were summarized as part of Theme 2 in this study defended positions that were in direct opposition to those adopted by the Ethics Committee of the German Federal Ministry of Transportation (BMDV, 2017). As the world’s first governmental guideline issued on the topic of AVs and collision scenarios, its propositions are significant for our discussions herein. In Rule 2, the German guideline expressly rejects the notion of sacrificing innocent people for the benefit of other potential victims in an AV collision scenario: “protecting people takes precedence over all other utilitarian considerations” (BMDV, 2017, pg. 10). The guideline offers little explanation for this stance and simply states that the aim of an AV is “to reduce damage or even completely avoid it.” It is interesting to note that some would argue that the German guideline against utilitarian considerations does not rule out all calculations of risk (Geisslinger et al., 2021; Luetge, 2017). The objective of the guideline is to balance risk and reduce harm “until it is completely prevented” (BMDV, 2017, pg. 2). Therefore, risk assessment and harm avoidance are at the heart of the guideline.

The participants of our study who were inclined toward utilitarian positions, albeit in nuanced ways, were directly in opposition to this German position. However, it may be argued that the cohort of participants presented in Theme 2 of our results may affirm the broader principles contained in the German guideline. According to this cohort, the rules of the road were the most important value to be upheld, but there were also objections and nuances that were considered important. For example, participant 41 felt that the road was a public space and the public should therefore be allowed to cross the road at any point without having their lives forfeit. This participant outright rejected the notion of a “wrong” side of the road and noted that pedestrians were innately free (even if the law says otherwise) to be on the road wherever they wanted. The other participants vehemently rejected this proposition. Some participants of the cohort representing Theme 2 argued that the rules of the road superseded all other considerations, including utilitarian factors. These participants argued that one cannot simply ignore the rules of the road to save more lives. Here too, the nuances of the participants were important. For some experts, such as participants 11 and 16, failure to obey the rules might result in unforeseen situations that could have more serious consequences. Thus, it was a matter of safety that an AV obeys the rules of the road even at the cost of more lives.

### 4.2 Responsibility of disobeying the rules

The notion that obeying the rules is of paramount importance may be connected to the responsibilities of road users. For example, participants 5 and 6 argued that if a pedestrian broke the rules of the road, then they would bear all responsibility for collisions involving their death. That is to say that the manner in which an individual interacted with the rules (upholding or breaking them) had a direct bearing on the consequence of a collision. Thus, although participant 41 rejected the idea of regulating the road for pedestrians as “unethical,” the other participants felt that pedestrians who failed to obey the rules of the road bore the responsibilities of the consequences. Here, there was a sense among the participants that risk and responsibility are intimately connected; hence, in the present cohort of research participants, the basic principles of the German guideline were affirmed. The German guideline states that in instances where pedestrians bring risk into a traffic situation (such as crossing the street when the light is red), the pedestrians should be assigned more risk and implicitly bear certain consequences (BMDV, 2017; Luetge, 2017).

On a similar note, participant 36 argued that an AV should not disobey the rules of the road by moving to the “wrong” side of the road or mount the pavement as this would add risk to pedestrians who had the right to be where they were. This sense of responsibility for either obeying or not obeying the rules was strongly present among those participants who argued that using an AV entailed a sense of responsibility and its consequences, even against utilitarian considerations. For example, participant 45 felt that even children on a bus (by virtue of their parents putting them on a bus) were subject to the risks of the road and should therefore bear the associated responsibilities and consequences.

### 4.3 Complexities of self-preservation

Although it is true that research has indicated that large sections of the public believe a utilitarian AV would be the most moral (Bonnefon et al., 2016), the question of self-sacrifice or self-preservation is highly complex (Frison et al., 2016). The public tends to have a preference toward riding in AVs that would protect them at all costs (Sui, 2023; Frison et al., 2016). This may be way Google has described a patent by which an AV might position itself in a lane so as to decrease its own risk (Dolgov and Urmson, 2014), while Mercedes-Benz has announced that it would design AVs to prioritize the safety of occupants over other road users (Morris, 2016; Taylor, 2016). Presumably, these decisions were based on the commercial interests of Waymo and Mercedes-Benz as it would be harder to sell cars that could deprioritize customers in collision scenarios. This concept is known as the selfish or egoistic AV (Liu and Liu, 2021; Morris, 2016; Sui, 2023), for which some researchers have argued that it would be paradoxically better to program cars to prioritize occupants so that more AVs will be sold, thereby reducing road deaths and protecting greater numbers of lives (Bonnefon et al., 2016).

This egoistic drive for self-preservation was also noted in a small number of participants in our study. In the case of participant 25, this idea was directly linked to an egoistic concept of a human driver; that is to say, participant 25 felt that human drivers would be egoistic by nature, so AVs should be programmed to act like humans would. However, other researchers have argued this controversial point (Hang et al., 2021; Shaw et al., 2020). In the case of participant 46, this idea was linked to the commercial interests of the manufacturer, as indicated in the aforementioned paradox. However, even as these participants claimed to be egoistic, it is not clear whether they would actually promote such a purely egoistic design or even buy an egoistic AV.

Mercedes-Benz has received significant public criticism for its egoistic stance (Leben, 2017), with some experts arguing that the public would not feel comfortable purchasing an egoistic AV. Some researchers (Sui, 2023; Ju and Kim, 2024; Zhu et al., 2022) demonstrated through quantitative surveys involving large numbers of participants that there are significant disparities between the public’s moral preferences over AV design and purchase decisions. In the study by Sui (2023) involving 460 participants, the subjects were reluctant to sacrifice themselves to save five others but were also reluctant to sacrifice five others to save a single pedestrian; here, the researchers concluded that people attempt to balance competing moral considerations rather than subscribe to a single principle; in this research, the public were egoistic when the scenarios threatened themselves as the occupants of an AV but were utilitarian when only pedestrians were threatened. The research participants were more accepting of algorithms designed with a “hybrid” approach, whereby self-sacrifice was limited only to their selves as occupants, while a utilitarian design was applied to scenarios involving pedestrians only (Sui, 2023); these participants demonstrated a preference for both the design and purchase of such “hybrid algorithms.” Consequently, it is arguable that many of the experts who upheld utilitarian positions in our research may do so in pedestrian-focused scenarios but still hold to an egoistic position when they perceive themselves as the occupants. In other words, they favor a utilitarian framework when being objective and unbiased but abandon the objective framework for prudential and egoistic reasons when faced with such risk themselves.

### 4.4 Limitations

This study has several limitations, and we particularly note four such drawbacks. First, the methodology employed in this research lacks systematic prompts owing to the experimental ethics approach; in this approach, the participant responses were gently challenged by amending the parameters of the vignettes to elicit rich data on why the experts held certain opinions and what could inspire changes to these opinions. Since the original responses of each participant were unique, it was not possible to offer the same follow-up prompt to all participants. Therefore, it is not possible to analyze the relative importance of one feature of a character over another, such as determining whether the experts felt that larger numbers of affected persons are more important than obeying the rules. Nevertheless, a limited set of variable changes was selected to avoid entirely unique responses and the charge of nominalism (see Methodology section for more). This approach allowed maximum flexibility during the interviews while also eliciting rich data that were comparable across the participants.

Second, many of the participants were not English speakers, particularly the experts among the European population. Therefore, the grammar and vocabulary of some participants were not fluent. Nevertheless, all non-English-speaking participants worked in highly international environments, such as high-ranking universities. Hence, even if their English was not fluent at times, we do not believe that this impacted the underlying meaning of their responses significantly.

Third, owing to the nature of our recruitment process, the majority of participants in this research were primarily from a Western background. Although a small minority of the participants had non-Western backgrounds, such as Eastern or Middle Eastern researchers working in Switzerland, these particular experts may be considered westernized in that they had spent significant amounts of time in Western contexts. Therefore, it should be noted that the results are most likely applicable only to a Western context.

Fourth, an order bias may be present given that Vignette 1 was presented before Vignette 2 in almost all cases; it is possible that this order of presentation may affect the initial responses of the participants in Vignette 2. However, the intended neutral framing of Vignette 1 was a methodological choice that may have been affected had it been presented after Vignette 2 and its subsequent parameter changes. This remains a limitation of the present research given that it could induce a possible order bias. However, the authors feel that this influence would be minimal and would not decidedly affect the initial responses of the participants or their reasons for changing their opinions across the two vignettes.

## 5 Conclusion

This study presents our pioneering qualitative empirical research efforts into understanding the ethical opinions of experts who are directly involved in the development, deployment, and regulation of AVs on public roads. Our finding indicate that a large proportion of these experts hold a utilitarian perspective that is more nuanced than the simple belief that saving more lives is intrinsically more valuable. In many cases, the utilitarian views were associated with other factors, such as pragmatism; it is harder to predict the movements of groups of people versus an individual, and AVs should therefore be programmed to generally avoid groups of pedestrians for safety reasons. In addition, some participants tended to connect utilitarianism with a sense of the greater good for the community; that is to say that killing more people rather than fewer would have more of an impact, be more painful, and be more damaging to the community. Hence, they technically favored the minimization of disutility over maximization of utility. This theme was also present in the responses of other participants who felt that the personal characters of the persons involved were important in deciding who might live or die; the loss of a person of good character was believed to have a greater impact on the community than the loss of an individual of poor character.

Nevertheless, even as a large proportion of the participants held a utilitarian perspective as the reason for changing or not changing their initial opinions, a substantial number of experts in the cohort felt that obeying the rules was more important than utilitarian considerations. According to these participants, disobeying the rules could create unforeseen consequences and should be avoided at all costs even with the loss of more lives. Moreover, there was a sense among some members of this cohort that disobeying the rules may entail increased responsibility and consequences. This was in line with the German guideline on AVs, which stressed that users who bring added risk to the transport context should themselves be afforded such added risk (Luetge, 2017; Shaw et al., 2020). In simple terms, this means that persons who bring cars onto the road that can increase the risk of death and injury in the transportation context should bear such risk themselves. This sense of responsibility in the road context applies equally to AV users. To some participants, the risk and consequences of collision scenarios went hand in hand with the risk brought by AVs themselves. An AV user would personally bear responsibility for the consequences of using AVs on public roads, including in collision scenarios. This inherent responsibility was seen as both direct and indirect responsibility, where children could be held responsible for the decisions of their parents to allow them to use an AV.

Finally, the findings presented herein demonstrate that there are experts who affirm egoistic designs for AVs. This egoistic view is associated with the understanding that it may be more difficult to sell a utilitarian AV. Although this view mirrored some opinions in literature, it was also in opposition with other empirical research indicating that the opinions of the general public on the salability of an AV is far more complex than a simple egoistic or utilitarian perspective.

The results of this study are significant in that they provided insights into the ethical thinking of individuals who have direct influences on how AVs are deployed on public roads and how they may/should behave in collision scenarios. In particular, the findings reported herein provide rich data on the underlying ethical framework of such experts, including strongly held beliefs (that do not change under challenge) and/or the principles that experts believe merit changes in their opinions. The findings indicate that expert opinions are varied, nuanced, and wide-ranging, with very little observable homogeneity. If AVs are to be programmed such that they are accepted by the public, further research efforts are needed to identify not only the beliefs of experts but also how their ethical frameworks can be shaped in coherently ethical ways that are simultaneously acceptable to the public. Elsewhere, we have also considered the need for states themselves to provide ethical frameworks including both the public and others as stakeholders (Milford et al., 2024).

## Data Availability

The raw data supporting the conclusions of this article will be made available by the authors, without undue reservation.
